# Draft Genome Sequence of a Lactobacillus fermentum Strain Isolated from Domestic Sewage in Kerala, India

**DOI:** 10.1128/MRA.00713-20

**Published:** 2020-07-16

**Authors:** Pradeesh Babu, Archana Palillam Veedu, Vidhya Prakash, Megha Prasad, Amrita Salim, Ajith Madhavan, Bipin G. Nair, Sanjay Pal

**Affiliations:** a School of Biotechnology, Amrita Vishwa Vidyapeetham, Kollam, Kerala, India; University of Maryland School of Medicine

## Abstract

We report the draft genome sequence of a putative probiotic strain, Lactobacillus fermentum ASBT-2, isolated from domestic sewage in Kerala, India. The strain showed probiotic properties (tolerance to low pH and bile salts, binding to host matrix) and reduced the coliform count by 90% in a biofilter used to treat wastewater.

## ANNOUNCEMENT

We have developed a microbiome engineering tool to treat wastewater and food with potential probiotic strains and bacteriophages isolated from domestic sewage. Lactobacillus fermentum is recognized as a potential probiotic strain with antimicrobial, antioxidative, and cholesterol reduction properties (1–5). The organism was isolated from domestic sewage in Kerala, India, cultured in selective medium, De Man, Rogosa and Sharpe agar (MRS) (6), and confirmed with 16S rRNA gene ribotyping (7).

Genomic DNA was extracted using the phenol-chloroform method (8). The paired-end sequencing library was prepared using the TruSeq Nano DNA library prep kit with an average library size of 478 bp. The Illumina HiSeq platform was used for sequencing (9, 10) the paired-end library, with a read length of 2 × 150 bp. Both quantity and quality checks of the amplified library were performed in a Bioanalyzer 2100 instrument (Agilent Technologies) using a high-sensitivity DNA chip per the manufacturer’s instructions. High-quality (5.63 Gb) data, obtained after filtering the reads through Trimmomatic (v0.30) with a quality value (QV) of >20, were used for assembly. All the software settings used were under the default parameters unless otherwise mentioned. *De novo* assembly of paired-end reads was performed using Velvet v1.2.10. (11), and assembly was optimized with a kmer value of 121. The gaps of the assembled scaffold were filled using Gapcloser v1.12 (12). The total number of reads was 37,902,034, and the details of the assembled genome are listed in Table 1. tRNAscan-SE v1.3.1 was used for identification of probable tRNA genes (13). RNAmmer v1.2 was used for rRNA gene identification (14), which yielded a total of five 5S rRNAs and one 16S rRNA.

**TABLE 1:** Summary of the sequencing details of ASBT-2

Characteristic	Value
Total no. of contigs	74
Total no. of scaffolds	64
Total genome size, including gaps (Ns) (bp)	2,028,463
Total genome size, without gaps (Ns) (bp)	2,027,369
Contig *N*_50_ (bp)	70,502
Scaffold *N*_50_ (bp)	70,502
Avg scaffold length (bp)	31,695
Maximum scaffold length (bp)	146,327
GC content (%)	51.89
No. of tRNAs decoding standard 20 amino acids	54
No. of 5S rRNAs	5
No. of 16S rRNAs	1
Total no. of protein-coding genes	2,019
Total gene length (bp)	1,748,088
Maximum gene length (bp)	4,473
Avg gene size (bp)	865

The 64 scaffolds obtained from *de novo* assembly were subjected to gene prediction using Prodigal v2.6.3 (15), which resulted in the identification of 2,019 coding sequences. The predicted proteins of genes were subjected to a similarity search against NCBI’s nonredundant (nr) database using the BLASTP algorithm. Out of 2,019 predicted proteins, 1,989 got a hit in the NCBI database; the remaining 30 were novel proteins. Simultaneously, all the 2,019 proteins were searched for similarity against the UniProt, COG, and Pfam databases using BLASTP with an E value threshold of 1e^−5^.

### Data availability.

This whole-genome shotgun project has been deposited at GenBank under BioProject number PRJNA639667, SRA accession number SRR12020697, and BioSample accession number SAMN15244744.
